# Drug Pipeline for MASLD: What Can Be Learned from the Successful Story of Resmetirom

**DOI:** 10.3390/cimb47030154

**Published:** 2025-02-27

**Authors:** Elizabeta Knezović, Marija Hefer, Suzana Blažanović, Ana Petrović, Vice Tomičić, Nika Srb, Damir Kirner, Robert Smolić, Martina Smolić

**Affiliations:** 1 Faculty of Dental Medicine and Health, Josip Juraj Strossmayer University of Osijek, 31000 Osijek, Croatia; elizabeta.jakovec@gmail.com (E.K.); mhefer@fdmz.hr (M.H.); sblazanovic@fdmz.hr (S.B.); anapetrovic@fdmz.hr (A.P.); vtomicic@fdmz.hr (V.T.); nsrb@fdmz.hr (N.S.); damir.kirner@gmail.com (D.K.); rsmolic@fdmz.hr (R.S.); 2 Clinical Institute of Translational Medicine, University Hospital Osijek, 31000 Osijek, Croatia

**Keywords:** MASLD, MASH, resmetirom, THR-β agonists

## Abstract

Metabolic dysfunction-associated steatotic liver disease (MASLD) and its progressive form, metabolic dysfunction-associated steatohepatitis (MASH), represent a growing global health problem linked to obesity, insulin resistance, and dyslipidemia. MASLD often leads to fibrosis, cirrhosis, and hepatocellular carcinoma. Currently, therapeutic options are limited, emphasizing the need for novel, targeted pharmacological interventions. Resmetirom, a selective thyroid hormone receptor beta (THR-β) agonist, offers a promising approach by specifically enhancing hepatic metabolism while minimizing systemic effects. Clinical trials have demonstrated its capacity to reduce hepatic triglyceride accumulation and improve lipid profiles. Early- and advanced-phase studies, including the MAESTRO program, highlight significant reductions in hepatic fat content and favorable impacts on noninvasive biomarkers of fibrosis with minimal side effects. This review highlights evidence from pivotal studies, explores resmetirom’s mechanism of action, and compares its efficacy and safety with other emerging therapeutic agents. While resmetirom marks a breakthrough in non-cirrhotic MASH management, further long-term studies are essential to fully evaluate its clinical benefits and potential regulatory approval for broader use in MASLD and MASH.

## 1. Introduction

Metabolic dysfunction-associated steatotic liver disease (MASLD) and metabolic dysfunction-associated steatohepatitis (MASH) represent a significant global health problem. MASLD encompasses a spectrum of liver disorders characterized by hepatic steatosis, insulin resistance, obesity, dyslipidemia, and hypertension, often progressing to fibrosis, cirrhosis, and hepatocellular carcinoma [1,2,3]. MASLD closely parallels the global obesity epidemic and is strongly associated with metabolic syndrome. Approximately 90% of obese individuals, 60% of those with type 2 diabetes, and 50% of people with dyslipidemia develop MASLD [1,2,4,5]. Notably, the relationship is bidirectional, as up to 75% of individuals with diabetes have MASLD, and those with MASLD face an increased risk of developing diabetes. Furthermore, MASLD is frequently accompanied by lipid-profile abnormalities, such as elevated triglycerides and low HDL cholesterol, which exacerbate liver damage [5,6,7]. In obese individuals, the limited capacity of expanded adipose tissue to store lipids leads to excessive lipid accumulation, primarily in the form of triglycerides within hepatocytes. These lipids originate mainly from inappropriate dietary choices (15%), the enhanced lipolysis of triglycerides from adipose tissue (60%), and de novo lipogenesis in the liver (25%) driven by dietary sugars, glucose, and fructose [6,8]. As obesity and metabolic syndrome become increasingly prevalent, MASLD is expected to surpass other causes of chronic liver disease worldwide.

Starting from the accumulation of lipids in the liver (steatosis) caused by an imbalance between the intake and consumption of free fatty acids, MASLD progresses to MASH [9]. The enhanced production of reactive oxygen species due to steatotic changes promotes oxidative stress and the inflammation and destruction of hepatocytes, resulting in metabolic dysfunction and progression to MASH [9,10]. Epigenetic and epigenomic modifications, such as DNA and RNA methylation, further exacerbate the condition’s complexity. These modifications regulate key genes involved in lipogenesis, fatty-acid oxidation, and inflammation. Consequently, MASH can progress even further to liver fibrosis, cirrhosis, or potentially hepatocellular carcinoma if left untreated [11,12].

The disease’s pathophysiology involves complex interactions between lipid metabolism dysregulation, oxidative stress, inflammation, and genetic predispositions. Genetic variants, notably *PNPLA3 I148M* and *HSD17B13*, influence individual susceptibility. *PNPLA3 I148M* increases fibrosis risk, whereas *HSD17B13* offers protective effects by reducing inflammation [13,14].

Although there are several promising treatments in development, such as siRNA technology for *HSD17B13*, it should be noted that their availability and wider use in clinical practice are still limited and are in the research phase. In addition, the gut microbiome has been identified as a key factor in the development of MASLD [14]. However, therapies such as microbiome transplantation are still in the experimental phase. Currently, resmetirom (Rezdiffra™) is the only drug specifically approved by the US Food and Drug Administration (FDA) for the treatment of non-cirrhotic MASH, which means that there is a large scope for potential progress in the treatment of other liver disorders [15,16,17].

Lifestyle interventions focusing on weight loss remain a foundation for MASLD management. However, adherence is challenging, highlighting the urgent need for effective pharmacotherapies. Current treatments or dietary supplements for less advanced stages of MASLD, such as metformin, vitamin E, green tea catechins, and pioglitazone, have limited efficacy or adverse side effects [18,19,20,21,22,23]. Non-therapeutic interventions such as drinking water low in bicarbonate, sulfate, calcium, magnesium, and sodium have also been studied in patients with steatotic liver disease and have shown modest modulating properties as assessed by lower and upper gastrointestinal symptom-assessment questionnaires [24].

Among emerging therapies, resmetirom has garnered significant attention. Resmetirom targets key metabolic pathways within the liver, offering a promising approach for broader MASLD and MASH treatment [15,25]. However, the long-term safety and efficacy of resmetirom have not yet been fully evaluated in longitudinal clinical trials. By targeting pathophysiological processes within the liver, thyroid hormone receptor beta (THR-β) agonists such as resmetirom are essential for the treatment of metabolic dysfunction associated with hepatic steatosis [15,25,26,27].

## 2. Resmetirom: A Breakthrough in MASLD/MASH Management

### 2.1. Mechanism of Action

THR-β agonists have great potential for the treatment of MASLD, MASH, and related sequelae through their selective effect on the liver by reducing systemic side effects, including thyrotoxicosis. Targeted therapy with minimal side effects on the heart and other organs has been made possible by the discovery of selective THR-β agonists such as GC-1 (sobetirome), KB-2115 (eprotirome), and MGL-3196 (resmetirom). These drugs have shown encouraging effects on reducing liver steatosis, improving the lipid profile and reducing inflammation [28,29,30].

While THR-α is linked to negative effects on the heart and bones, THR-β, which is predominantly expressed in the liver, plays a critical role in lowering cholesterol levels and enhancing liver metabolism. This functional distinction has driven the development of selective THR-β agonists, designed to minimize systemic adverse effects while delivering targeted liver-specific metabolic advantages [31]. Resmetirom exerts its therapeutic effects by selectively activating THR-β in hepatic tissue, sparing THR-α receptors associated with cardiovascular and bone-related side effects. THR-β activation enhances mitochondrial fatty-acid oxidation, reduces lipogenesis, and promotes cholesterol efflux. By upregulating genes involved in lipid metabolism, such as carnitine palmitoyltransferase 1 (*CPT-1*), and downregulating sterol regulatory element-binding protein 1c (*SREBP-1c*), resmetirom effectively decreases hepatic triglyceride accumulation [15,28,29,30,32,33,34,35]. It also suppresses pro-inflammatory and pro-fibrotic mediators, including transforming growth factor-beta (TGF-β), and reduces lipogenesis by downregulating lipogenic genes encoding fatty acid synthase (FASN) and acetyl-CoA carboxylase 1 (ACC1), both often elevated in MASLD [32,36].

Resmetirom, when administered in doses of 80 mg and 100 mg per day, has demonstrated a significant positive impact on liver health, particularly in improving liver fibrosis. Clinical studies have provided clear evidence supporting its effectiveness, demonstrating notable reductions in low-density lipoprotein (LDL) cholesterol levels. Specifically, patients receiving an 80 mg daily dose experienced a reduction in LDL cholesterol by 13.6%, while those on the higher 100 mg dose achieved an even greater reduction of 16.3% [15,37].

Unlike older thyroid hormone analogs, resmetirom minimizes systemic effects. Its targeted action on liver metabolism reduces de novo lipogenesis, improves insulin sensitivity, and lowers serum lipids. Clinical data confirm its ability to reduce liver fat without adversely impacting weight, glucose homeostasis, or cardiovascular markers. Also, according to clinical trials, resmetirom’s beneficial effects on circulating lipids could help reduce the risk of major cardiovascular adverse events in patients with MASLD/MASH [38,39,40].

However, resmetirom should not be used in patients with decompensated cirrhosis, as moderate to severe hepatic impairment may increase the risk of adverse events due to an increase in C_max_ and overall drug exposure over time [41]. Moreover, given that resmetirom is primarily metabolized by cytochrome P450 (CYP) 2C8, potential drug–drug interactions with CYP2C8 inhibitors, such as repaglinide, clopidogrel, and statins, should be carefully considered before administration [42].

### 2.2. Early Clinical Trials

Phase 1 trials established resmetirom’s pharmacokinetics, safety, and lipid-lowering efficacy. Single- and multiple-dose studies demonstrated reductions in LDL cholesterol, triglycerides, and non-HDL cholesterol. Importantly, resmetirom was well tolerated, with mild gastrointestinal symptoms as the most common side effects. No significant alterations in thyroid function tests or cardiac markers were observed [43,44].

Some of the first-in-human studies of resmetirom were randomized, double-blind, placebo-controlled phase 1 trials evaluating its safety, tolerability, and pharmacokinetics. A total of 72 healthy participants received single oral doses ranging from 0.25 mg to 200 mg. A multiple-dose study followed, involving 48 participants over 14 days with doses between 5 mg and 200 mg. Resmetirom significantly reduced LDL-C, non-HDL-C, ApoB (up to 30%), and triglycerides (up to 60%), showing promise for dyslipidemia treatment. The drug was well tolerated with no serious adverse effects, though higher doses caused mild reductions in free thyroxine (T4). This supports resmetirom’s potential as a safer alternative to earlier thyroid analogs [30,45]. However, long-term monitoring is necessary to assess potential risks associated with thyroid, gonadal, or bone diseases [40].

### 2.3. Phase 2 Trial Results

The pivotal phase 2 trial investigated resmetirom in patients with biopsy-confirmed MASH and hepatic fat content ≥ 10%. Over 36 weeks, resmetirom (80 mg daily) achieved a 32.9% reduction in hepatic fat compared to 10.4% with placebo. Secondary outcomes showed improvements in lipid markers, liver enzymes, and fibrosis biomarkers. Resmetirom lowered atherogenic lipids, small dense LDL, and large VLDL, reducing cardiovascular risks. Furthermore, liver biopsies revealed a reduced NAS score, fibrosis-stage improvement in 61% of patients, and fibrosis resolution in 56%. The treatment was well tolerated, with diarrhea and nausea as the most common side effects [46].

Another trial investigated whether higher doses or longer treatment with resmetirom could improve MASH and fibrosis outcomes in patients with incomplete responses from the initial study. Some resmetirom-treated patients had shown partial improvements, but those on lower doses (60 mg) had less MASH resolution than those on 80 mg or 100 mg. By 36 weeks, 53% of resmetirom-treated patients achieved a 2-point NAS reduction, and 17.6% had fibrosis improvement, compared to 14.3% and 0% in the placebo group. Resmetirom also significantly reduced liver fat, liver enzymes, fibrosis markers, and inflammation markers, highlighting its potential to target fibrosis and liver inflammation [47].

### 2.4. Comprehensive Phase 3 Trials

Encouraged by these promising phase 2 results, the phase 3 MAESTRO clinical program was initiated to assess resmetirom in larger, more diverse populations. These trials aim to evaluate the safety, efficacy, and long-term outcomes of resmetirom in MASH patients with varying degrees of liver disease, including those with metabolic risk factors such as obesity, type 2 diabetes, hypertension, and dyslipidemia [34].

The MAESTRO program, comprising multiple trials, aimed to validate resmetirom’s long-term efficacy and safety across diverse MASLD populations.

The MAESTRO-NAFLD-1 trial was a 52-week, randomized, double-blind, placebo-controlled trial involving 1400 patients at 80 US sites. This trial evaluated the safety and efficacy of resmetirom (80 mg and 100 mg) in MASLD and presumed MASH. This study also included treatment for non-cirrhotic MASH and MASH cirrhosis, providing valuable data on resmetirom’s safety in patients taking thyroid medications [48]. The MAESTRO-NAFLD-OLE extends the MAESTRO-NAFLD-1 study by providing up to 2 years of treatment for patients who initially participated in the study. After 12 weeks of double-blind treatment, all patients were switched to receive 100 mg dose of resmetirom. This extension involved 700 patients from the parent trial’s non-cirrhotic MASH and MASH cirrhosis [34]. Subsequently, MAESTRO-NASH involved a 54-month trial that targeted non-cirrhotic MASH with advanced fibrosis. Preliminary data indicated a 29.9% MASH resolution rate with resmetirom versus 9.7% with placebo, and fibrosis improvement in 25.9% of cases [48].

Together, these phase 3 trials form a crucial part of the regulatory pathway for resmetirom’s potential approval as a treatment for MASH, as the trials focused on evaluating both short-term biomarkers and long-term clinical outcomes like mortality, liver decompensation, and progression to cirrhosis. In addition to adult MASH patients, resmetirom has shown potential for treating MASLD in children with obesity, with encouraging results in improving steatohepatitis and fibrosis in up to 36% and 27% of patients, respectively [49].

As of 21 October 2024, the trial by Madrigal Pharmaceuticals has completed enrollment for the treatment of patients with compensated MASH cirrhosis. While specific results are pending, prior data indicate resmetirom’s antifibrotic properties, supporting its potential benefits. Madrigal Pharmaceuticals has made significant advancements with its resmetirom clinical trial program. The recent completion of enrollment for the MAESTRO-NASH OUTCOMES trial could provide a positive outcome, leading to resmetirom becoming the first approved therapy for this high-risk population while supporting full regulatory approval for broader MASH indications [50].

## 3. Comparison with Other Therapeutic Approaches

MASLD is linked to Type 2 Diabetes Mellitus (T2DM), obesity, and other metabolic disorders, prompting the exploration of different drugs, including antidiabetic agents for therapy. Recent research has contributed to the advancement of several drugs into phase 2 and 3 clinical trials for the treatment of MASLD and MASH, as highlighted in the studies discussed below [51,52].

**Figure 1:**
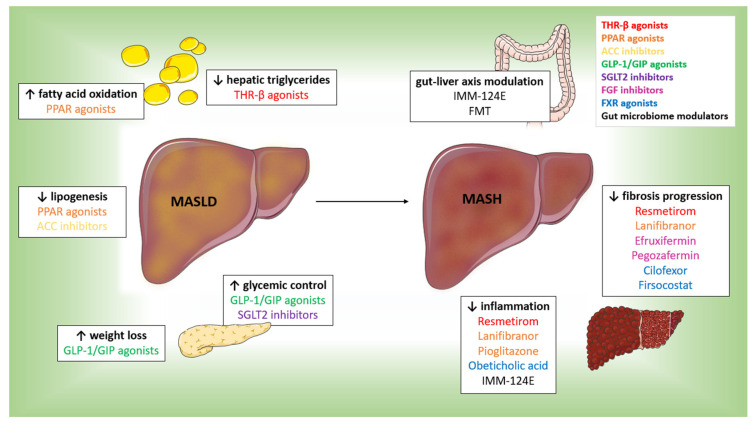
Pharmacological interventions in MASLD and MASH progression. Figure created with Servier Medical Art, https://smart.servier.com/ (accessed on 17 February 2025).

Figure 1 highlights the impact of these drugs on the progression of both MASLD and MASH, emphasizing the specific drugs that demonstrated beneficial effects on inflammation and fibrosis progression.

### 3.1. THR-β Agonists

Along with resmetirom, sobetirome and eprotirome have shown positive effects on hepatic steatosis, reducing hepatic triglycerides in MASLD and insulin-resistant ob/ob mice. However, these improvements did not consistently enhance insulin sensitivity or glycemic control, with lower doses of sobetirome impairing glycemia while higher doses improved it, despite all doses equally reducing liver triglycerides [53].

It was also noted that sobetirome reduced hepatic triglycerides by 75% but caused fasting hyperglycemia, hyperinsulinemia, and reduced hepatic insulin sensitivity due to increased endogenous glucose production (EGP) from enhanced lipolysis. In contrast, eprotirome prevented hepatic steatosis without affecting EGP or fasting glycemia but impaired peripheral glucose disposal by lowering skeletal muscle GLUT4 levels despite normal insulin signaling [54].

VK2809, another liver-selective THR-β agonist, shares resmetirom’s mechanistic focus but remains in earlier developmental stages. Preliminary data suggest potent lipid-lowering effects with minimal systemic impact. VK2809 demonstrated a reduction in liver lipid content in MASLD patients, followed by evaluation in a 52-week phase 2b trial [55,56].

The phase 2b clinical trial for VK2809, known as the VOYAGE study, has been completed by Viking Therapeutics, Inc. (San Diego, CA, USA) for the evaluation of VK2809 in patients with biopsy-confirmed MASH. The study successfully achieved its primary endpoint, with patients receiving VK2809 experiencing statistically significant reductions in liver fat content by 37% to 55% after 52 weeks of treatment. The treatment was well tolerated, with the majority of adverse events reported as mild or moderate gastrointestinal-related adverse events [56,57,58,59].

Therefore, along with VK2809, resmetirom’s safety profile remains favorable. Mild gastrointestinal symptoms, including diarrhea and nausea, are transient and dose dependent. Unlike other drugs, resmetirom does not cause weight gain or fluid retention. Its selective action on hepatic THR-β avoids the thyrotoxic and cardiometabolic complications linked to non-selective thyroid analogs [53,54,60].

### 3.2. GLP-1 and GIP Receptor Agonists

Glucagon-like-peptide-1 (GLP-1) receptor agonists, approved for diabetes and obesity, were among the first tested in MASLD, with successful phase 2 trials progressing to phase 3. New combinations with glucagon or glucose-dependent insulinotropic peptide (GIP) receptor agonists have shown improvements in weight, insulin resistance, and liver parameters, although the direct effects of both agonists on MASLD remain debated [61,62]. GLP-1 and GIP receptor agonists are increasingly explored for MASLD. While effective in weight reduction and glycemic control, their impact on fibrosis remains inconsistent.

Semaglutide (Ozempic^®^), one of the approved antidiabetic agents, generally showed promising MASH resolution rates but limited fibrosis improvement [61,63,64,65,66]. A phase 2 trial showed that semaglutide led to higher MASH resolution rates compared to placebo, though it did not significantly improve fibrosis [67]. In patients with MASH and cirrhosis, semaglutide showed no significant improvement in fibrosis or MASH resolution [68]. Similarly, liraglutide has shown a reduction in weight, hepatic steatosis, and hepatocellular apoptosis in MASLD patients, but its benefits were not sustained after discontinuation [69]. Dual GLP-1 and GIP agonists, such as tirzepatide, have shown more profound effects, including significant weight reduction in the SURMOUNT study [70]. A SURPASS-3 substudy found that tirzepatide significantly reduces liver fat content compared to insulin degludec [71]. Retatrutide, a GIP, GLP-1, and glucagon receptor triple agonist, led to a 24.2% mean weight reduction after 48 weeks in a phase 2 trial, with phase 3 trials underway. This study demonstrated that retatrutide produced significant, dose-dependent reductions in body weight over 48 weeks, with the highest reduction of 24.2% in the 12 mg group compared to 2.1% in the placebo group. Gastrointestinal adverse events were the most common and dose related, along with an increase in the heart rate at 24 weeks, but were generally mild to moderate [72].

### 3.3. SGLT2 Inhibitors

Sodium-glucose cotransporter 2 (SGLT2) inhibitors, including dapagliflozin and empagliflozin, improve glycemic control and cardiovascular outcomes. However, their primary effect on hepatic fat reduction is modest, and fibrosis data are limited [73].

SGLT2 inhibitors (canagliflozin, dapagliflozin, empagliflozin, and ertugliflozin) are approved for T2DM and improve cardiovascular risks and hepatic health. Empagliflozin reduces liver fat and improves ALT levels in T2DM patients with MASLD [74]. Along with empagliflozin, dapagliflozin improves liver steatosis but attenuates fibrosis while reducing serum alanine aminotransferase and γ-glutamyltranspeptidase levels, especially in patients with significant fibrosis [75]. Dapagliflozin has also shown improvements in hepatic fat content, body weight, and glycemic control [76,77]. Although SGLT2 inhibitors are recognized for reducing hepatic lipid content and fibrosis through multiple mechanisms, the use of dapagliflozin is consistently associated with serious renal-related adverse effects [78,79].

### 3.4. PPAR Agonists

Peroxisome proliferator-activated receptor (PPAR) agonists, including pioglitazone, rosiglitazone, and lanifibranor, play a key role in regulating lipid metabolism and reducing inflammation. Pioglitazone effectively resolves MASH but induces weight gain. On the other hand, lanifibranor, a pan-PPAR agonist, demonstrates promising anti-fibrotic effects, warranting further research [80,81,82,83,84].

PPAR agonists are known to regulate genes involved in metabolism, making them targets for MASH therapies. Lanifibranor, a pan-PPAR agonist, significantly reduced SAF-A scores in a phase 2b trial, supporting phase 3 trials [80]. Another study found that lanifibranor significantly improves insulin resistance in hepatic, muscle, and adipose tissues, effectively reducing intrahepatic triglyceride content and improving key cardiometabolic risk factors in patients with MASLD and T2D [85]. Saroglitazar, a PPAR-α/γ agonist, improved histological and metabolic markers in MASH [81].

Thiazolidinediones (TZDs), such as pioglitazone and rosiglitazone, improve insulin sensitivity by activating PPARγ nuclear receptors. Pioglitazone has demonstrated significant therapeutic benefits in patients with MASH, with 58% achieving the primary outcome and 51% experiencing MASH resolution [82]. Pioglitazone, also demonstrating PPARα activity, can improve liver inflammation and steatosis [83]. Similarly, rosiglitazone has shown an improvement in liver steatosis and normalized transaminase levels in MASH patients, with nearly half showing a 40% reduction in liver fat [84].

### 3.5. FGF and ACC Inhibitors

Fibroblast growth factor 21 (FGF21) analogs, such as efruxifermin, and acetyl-CoA carboxylase (ACC) inhibitors show promise in reducing steatosis and fibrosis. Efruxifermin improves fibrosis and metabolic parameters, while ACC inhibitors target hepatic lipogenesis. Efruxifermin, an Fc-FGF21 analog, improved fibrosis and resolved MASH in a phase 2b trial [86]. Pegozafermin, a glycopegylated FGF21 analog, showed fibrosis improvement in a phase 2b trial, prompting phase 3 development [87]. Fibroblast growth factor 19 (FGF19) offers another therapeutic pathway by promoting cell proliferation and repair. Aldafermin, an engineered FGF19 analog, reduced liver fat and showed trends toward fibrosis improvement in a 24-week phase 2 study [88]. NGM282, another FGF19 analog, led to rapid reductions in liver fat in a phase 2 trial [89].

Allosteric inhibitors of acetyl-coenzyme A carboxylases (ACC1 and ACC2) suppress hepatic lipogenesis and improve steatosis, inflammation, and fibrosis. In phase 2a studies, the ACC1/2 inhibitor PF-05221304 reduced liver fat by 50–65%, with additional benefits when combined with PF-06865571 [90,91].

### 3.6. FXR Agonists

Farnesoid X receptor (FXR) plays a key role in metabolic regulation in MASLD and MASH, making it an important therapeutic target. Obeticholic acid (OCA), an FXR agonist, is known to improve insulin sensitivity and reduce inflammatory markers in MASLD patients, although longer-term studies are needed [92]. Other FXR modulators like EDP-305 and MET409 have shown improvements in ALT and liver fat, with MET409 demonstrating a better side effect profile [93,94,95]. Another FXR agonist, tropifexor, has shown a reduction in ALT levels and liver fat but caused pruritus, while nidufexor reduced steatosis and inflammation in phase 2 trials [96,97].

Moreover, in a phase 2b trial, cilofexor and firsocostat showed significant improvements in NAS, liver biochemistry, and fibrosis markers compared to placebo in patients with F3-F4 fibrosis or compensated cirrhosis [98].

### 3.7. Gut Microbiome Modulators

Emerging research highlights the potential of gut–liver axis modulation as a therapeutic approach for MASH. Gut microbiome dysbiosis, a key factor in disease progression, increases gut permeability and triggers liver inflammation. Among promising adjunctive therapies, IMM-124E, a bovine colostrum-derived product, has been shown to significantly reduce AST and ALT levels in MASH patients after 24 weeks of treatment, although it did not affect liver fat content [99,100]. Additionally, fecal microbiota transplantation (FMT) is being explored, though clinical evidence for both therapies remains preliminary. Notably, IMM-124E has demonstrated a favorable safety profile, with no reported adverse effects [100,101].

The mechanism of action, advantages, and disadvantages of the previously mentioned therapeutic options are summarized in Table 1.

## 4. Conclusions

Resmetirom, being the first FDA-approved drug for non-cirrhotic MASH, represents a significant advancement in the treatment of MASLD and MASH, demonstrating efficacy in reducing hepatic fat and improving lipid profiles with mild side effects.

In this review, we have highlighted the current advancements and emerging therapeutic strategies for MASLD and MASH, with a focus on resmetirom. Resmetirom stands out as a promising agent for broader MASH indications due to its significant improvements in hepatic steatosis, fibrosis markers, and overall metabolic profiles, demonstrating a favorable safety profile with mostly mild gastrointestinal effects. VK2809, similarly effective in reducing liver fat and lipids, presents with minimal adverse effects, making it another promising agent for MASLD/MASH management. While resmetirom is further along in clinical development, long-term studies are also required to fully confirm its safety and sustained benefits.

While these selective THR-β agonists offer a great potential in the treatment of MASLD/MASH, several other agents targeting different pathways show varying degrees of efficacy and safety. Notably, GLP-1 and GIP receptor agonists improve metabolic parameters but exhibit inconsistent effects on fibrosis and are often associated with gastrointestinal side effects. SGLT2 inhibitors like dapagliflozin offer benefits in reducing hepatic lipid content, but some can carry the risk of renal-related adverse events. PPAR agonists, such as pioglitazone and rosiglitazone, improve insulin sensitivity and overall liver health but may lead to weight gain and cardiovascular risks, respectively. However, despite the progress in MASLD/MASH treatment, long-term studies are still needed to confirm the sustained safety and effectiveness of these therapies.

## Figures and Tables

**Table 1:** An overview of the therapeutic options for the treatment of MASLD and MASH.

Mechanism of Action	Agent	Advantages	Disadvantages	Reference
THR-β agonists	Resmetirom	Reduces hepatic fat, improves lipid profile, and lowers inflammation and fibrosis markers	Mild gastrointestinal symptoms (diarrhea, nausea), mild free T4 reduction at high doses	[30,32,36,38,45]
	Sobetirome	Reduces hepatic triglycerides, high doses improve hyperglycemia	Lower doses impair glycemia, causing fasting hyperglycemia, hyperinsulinemia, and reduced hepatic insulin sensitivity	[53,54]
	Eprotirome	Reduces hepatic triglycerides	Impairs peripheral glucose disposal by lowering skeletal muscle GLUT4 levels	[53,54]
	VK2809	Reduces hepatic lipogenesis, lowers lipid levels with minimal systemic impact	Mild or moderate gastrointestinal-related events	[55,56,57,58]
GLP-1 and GIP Receptor Agonists	Liraglutide	Reduces weight, hepatic steatosis, and hepatocyte apoptosis	Benefits not sustained after discontinuation	[69]
	Semaglutide	Enhances glycemic control, weight reduction, and reduces hepatic steatosis	Limited fibrosis improvement, nausea, vomiting	[61,63,64,67,68]
	Tirzepatide	Improves weight loss, insulin resistance, reduces liver fat	A dose-dependent increase in gastrointestinal-related events	[70,71,102]
	Retatrutide	Enhances weight loss and improves metabolic parameters	Higher-dose-related gastrointestinal events and dose-dependent increase in heart rate	[72]
SGLT2 Inhibitors	Empagliflozin	Improves ALT levels and glycemic control	N/A	[74]
	Dapagliflozin	Improves glycemic control, reduces liver fat, and serum alanine aminotransferase and γ-glutamyltranspeptidase levels	Acute kidney injury	[74,75,76,77,78,79]
PPAR Agonists	Lanifibranor	Significant histological improvement, fibrosis reduction and anti-inflammatory effects	Gastrointestinal-related events, peripheral edema, anemia, and weight gain	[80,81,82,83,85,103]
	Saroglitazar	Improves histological and metabolic markers in MASH	Gastritis, asthenia, and pyrexia	[81,104]
	Pioglitazone	Improves insulin sensitivity, reduces liver inflammation, 51% MASH resolution	Weight gain, fluid retention	[82,83,105]
	Rosiglitazone	Normalizes transaminase levels in MASH patients and reduces liver fat percentage	Increases LDL cholesterol, HDL cholesterol, and total cholesterol	[84,106]
FGF Inhibitors	Efruxifermin	Reduces fibrosis, improves metabolic parameters	Mild to moderate gastrointestinal events	[86,107]
	Pegozafermin	Reduces fibrosis, improves metabolic outcomes	Mild/moderate nausea and diarrhea	[87,108]
	Aldafermin	Reduces liver fat	Diarrhea	[88,109]
	NGM282	Significant reduction in hepatic steatosis	Injection site reactions, diarrhea, and nausea	[89]
ACC Inhibitors	PF-05221304	Significant reduction in liver fat	N/A	[90,91]
FXR Agonists	Obeticholic acid	Improves insulin sensitivity, reduces inflammatory markers	Pruritus, fatigue, constipation, abdominal distention	[92,93,110]
	EDP-305	Improves ALT and lipid profiles	High incidence of pruritus, dizziness, nausea, diarrhea	[95]
	MET409	Improves ALT and lipid profiles	N/A	[94]
	Tropifexor	Significantly reduces ALT, liver fat, and NAS	Pruritus	[96]
	Cilofexor	Improvements in liver parameters in patients with advanced fibrosis or compensated cirrhosis	Headache	[98,111]
	Firsocostat	Improvements in liver parameters in patients with advanced fibrosis or compensated cirrhosis	Nausea, diarrhea, and headache	[98]
Gut Microbiome Modulators	IMM-124E	Reduces inflammation, improves liver enzymes (AST and ALT), no reported adverse effects	No impact on liver fat noted in a single 24-week trial	[99,101]
