# Correction: Knezović et al. Drug Pipeline for MASLD: What Can Be Learned from the Successful Story of Resmetirom. *Curr. Issues Mol. Biol.* 2025, *47*, 154

**DOI:** 10.3390/cimb47120979

**Published:** 2025-11-25

**Authors:** Elizabeta Knezović, Marija Hefer, Suzana Blažanović, Ana Petrović, Vice Tomičić, Nika Srb, Damir Kirner, Robert Smolić, Martina Smolić

**Affiliations:** 1 Faculty of Dental Medicine and Health, Josip Juraj Strossmayer University of Osijek, 31000 Osijek, Croatia; elizabeta.jakovec@gmail.com (E.K.); mhefer@fdmz.hr (M.H.); sblazanovic@fdmz.hr (S.B.); anapetrovic@fdmz.hr (A.P.); vtomicic@fdmz.hr (V.T.); nsrb@mefos.hr (N.S.); damir.kirner@gmail.com (D.K.); rsmolic@fdmz.hr (R.S.); 2 Clinical Institute of Translational Medicine, University Hospital Osijek, 31000 Osijek, Croatia

There was an error in the original publication [1], A sentence was accidentally written in a way that it could possibly imply that resmetirom has been tested on children:

“In addition to adult MASH patients, resmetirom has shown potential for treating MASLD in children with obesity, with encouraging results in improving steatohepatitis and fibrosis in up to 36% and 27% of patients, respectively [49].”

A correction has been made to Paragraph 4 of the Section 2.4. Comprehensive Phase 3 Trials:

“In addition to adult MASH patients, resmetirom could show potential for treating MASLD in children with obesity, with encouraging results in improving steatohepatitis and fibrosis in up to 36% and 27% of 29 adult patients, respectively. However, there are currently no ongoing studies evaluating the effect of resmetirom in obese children with MASLD [49].

Despite promising results regarding the administration of resmetirom in adult patients with MASLD/MASH, there is a lack of clinical trials on its long-term safety in these patients. Also, absence of long-term studies on the progression of MASLD in children and adolescents remains an obstacle in developing pharmacotherapeutic approaches to pediatric MASLD. This highlights the need for further research assessing the long-term safety of resmetirom in adult patients, as well as the potential use of resmetirom in treating pediatric MASLD.”

The authors state that the scientific conclusions are unaffected. This correction was approved by the Academic Editor. The original publication has also been updated.
